# A refined spirometry dataset for comparing segmented (piecewise) linear models to that of GAMLSS

**DOI:** 10.1016/j.dib.2024.111062

**Published:** 2024-10-23

**Authors:** Gerald Stanley Zavorsky

**Affiliations:** Department of Physiology and Membrane Biology, Tupper Hall, Rm 4327, 1275 Med Sciences Drive, University of California, Davis, CA 95616, United States

**Keywords:** Biostatistics, Spirometry, Lung diffusing capacity, Restriction, Airway obstruction

## Abstract

Generalized Additive Models for Location, Scale, and Shape (GAMLSS) are widely used for developing spirometric reference equations but are often complex, requiring additional spline tables. This study explores the potential of Segmented (piecewise) Linear Regression as an alternative, comparing its predictive accuracy to GAMLSS and examining the agreement between the two methods. Spirometry data from nearly 16,600 patients, deemed Grade “A” and “B” acceptable from the NHANES 2007-2012 dataset, was analyzed. The dataset includes both nominal and scalar variables. Reference equations for forced expiratory volume in 1 s (FEV_1_), forced vital capacity (FVC), and the ratio (FEV_1_/FVC) were generated using GAMLSS (FEV_1_, FVC, FEV_1_/FVC), Segmented Linear Regression (FEV_1_, FVC) and multiple linear regression (FEV_1_/FVC). *K*-fold cross-validation was employed to compare prediction accuracy, using root-mean-square error (RMSE) and correlation coefficients. Agreement in classifying spirometric patterns (i.e. airway obstruction, restrictive spirometry pattern, mixed obstructive and restrictive disorder) was evaluated with the kappa statistic. This study uniquely compares the models by incorporating the lower limit of normal (LLN) using fitted z-scores of –1.645 or –1.96. The dataset is publicly available in SPSS (.sav) and .csv formats through the Mendeley Data repository.

Specifications Table

**Table:** 

Subject	*Health and* Medical Sciences
Specific subject area	Pulmonary and Respiratory Medicine*.*
Type of data	Filtered spirometry data (continuous data) included 16,596 subjects who could perform technically acceptable spirometry maneuvers. Age, weight, height, sex, and self-selected race/ethnicity are reported. As well, results of Generalized Additive Models of Location, Scale and Shape (GAMLSS) & segmented linear regression (SLR) modeling are provided in nominal formal. The data is in SPSS (.sav) and .csv format.
Data collection	Spirometry was performed in the standing position in those 6 to 80 years of age unless the participant was physically impaired. Data is from the 2007 to 2012 National Health and National Examination Survey (NHANES) dataset on spirometry measures. Excluded from testing were examinees that had current chest pain or a physical problem with forceful expiration, were taking supplemental oxygen, had recent surgery of the eye, chest, or abdomen, had a recent heart attack, stroke, tuberculosis exposure, or had recently coughed up blood. Adults with a personal history of detached retina or a collapsed lung and children with painful ear infections were also excluded.
Data source location	The National Health and National Examination Survey (NHANES) follows a complex, multistage probability sampling design aimed to be representative of the U.S. population. The survey is conducted in various locations across the United States, chosen randomly to ensure geographic and demographic diversity. Still, specific cities are not typically disclosed in public datasets to protect participant confidentiality. However, NHANES sampling is designed to cover a broad range of urban and rural areas across different regions in the United States, ensuring nationwide representation rather than focusing on specific cities. The data is collected by mobile examination centers (MECs) traveling to different locations yearly. https://wwwn.cdc.gov/nchs/nhanes/
Data accessibility	Repository name: Mendeley Data Data identification number: 10.17632/dwjykg3xww.1 Direct URL to data: https://data.mendeley.com/datasets/dwjykg3xww/1 The data are in two formats: A) SPSS (.sav) format. Once the file is open you will find the labels for each parameter under the “VARIABLE VIEW” tab. B) .csv format.
Related research article	G.S. Zavorsky, Debunking the GAMLSS myth: Simplicity reigns in pulmonary function diagnostics, Respir Med (2024) https://doi.org/10.1016/j.rmed.2024.107836

## 1 Value of the Data

• **Representative of the U.S. population**: NHANES follows a complex, multistage sampling process, ensuring that the data collected reflects a broad cross-section of the U.S. population. This makes the data highly representative and valuable for understanding lung function trends and disparities across different demographics, including age, race, and ethnicity.
• **High data quality and standards**: The spirometry data in NHANES meets strict technical standards set by the American Thoracic Society (ATS) and the European Respiratory Society (ERS). This ensures that the measurements for forced expiratory volume (FEV_1_), forced vital capacity (FVC), and the FEV_1_/FVC ratio are reliable and of high quality, suitable for clinical and research applications.
• **Extensive coverage of demographic groups**: The dataset includes spirometry data from over 16,000 participants from various racial and ethnic backgrounds (White, Black, Mexican American, Other Hispanic, and multi-racial). This allows for robust analysis of lung function differences across populations, contributing to developing race- or ethnicity-specific reference values, which are important for equitable healthcare.
• **Facilitates longitudinal and cross-sectional analysis**: The comprehensive nature of the dataset, collected over several years, makes it ideal for both cross-sectional studies and longitudinal analyses of lung function trends. It can be used to track how lung function changes with age, across different population groups, or in response to environmental and health factors.
• **Supports comparisons of modeling techniques**: This refined dataset has been used to compare different statistical modeling techniques, such as Generalized Additive Models for Location Scale and Shape (GAMLSS) and segmented linear regression (SLR) for the development of reference equations for use across the lifespan. This comparison allows researchers to explore complex and simplified lung function diagnostics approaches, ultimately improving the accuracy and accessibility of predictive models. Specifically, this dataset is unique in that includes the results of GAMLSS and SLR modeling using the lower limit of normal (LLN) for fitted z-scores of –1.645 or –1.96 for between model comparison.

## 2 Background

The NHANES 2007–2012 spirometry dataset is a key component of the National Health and Nutrition Examination Survey, a program designed to assess the health and nutritional status of the U.S. population through comprehensive, nationwide data collection. This dataset provides high-quality lung function measurements, including forced expiratory volume (FEV_1_) and forced vital capacity (FVC), meeting the rigorous technical standards set by the American Thoracic Society (ATS) and European Respiratory Society (ERS). It includes data from over 16,000 participants across diverse racial and ethnic groups, enabling the development of accurate reference values for pulmonary diagnostics. The data is highly representative of the U.S. population and has been used to evaluate the effectiveness of different statistical modeling techniques, such as Generalized Additive Models for Location Scale and Shape (GAMLSS) and Segmented (Piecewise) Linear Regression (SLR), for predicting lung function. The dataset's extensive demographic coverage and adherence to strict data quality protocols make it an invaluable resource for researchers and clinicians focused on respiratory health.

The current guidelines advocate for GAMLSS-derived reference equations, which require spline tables [[1], [2], [3], [4]]. However, previous research has shown that simpler SLR models – which do not require supplementary spline tables – provide similar predictive accuracies as GAMLSS for pulmonary diffusing capacity [5,6]. In those studies, predictive accuracies were defined by the root mean square error (RMSE), and obtained through repeated subsampling via the holdout method [5] or repeated *K*-fold cross-validation [6]. As such, it was thought that simple multiple linear regression or SLR would show comparable accuracies to GAMLSS when spirometric reference equations were developed.

This data article adds value by offering a comprehensive dataset that can be used for secondary analyses, comparative studies, and modeling efforts [7]. It enhances the primary findings of the published research study [8] by providing the raw data needed to validate and expand upon those results, thereby supporting broader research initiatives in pulmonary function diagnostics. Specifically, results from GAMLSS, SLR and multiple linear regression are provided in the dataset, and the R-code for GAMLSS and SLR replication is provided in the supplementary material section of this data-in-brief article.

## 3 Data Description

The refined dataset of 16,596 subjects is available in SPSS (.sav) format, which requires SPSS software to open [7]. However, there is an identical data file in .csv format [7]. Once the SPSS file is open, individuals can navigate to the “Variable View” tab. In this view, the “Label” column describes each parameter and its units (if applicable). For nominal variables, the “Values” column in the “Variable View” tab categorizes the variable. For example, “Sex” is a nominal variable; in the “Label” column, it is labeled as “Sex” (male or female). In the “Values” column, the coding is provided as 0 = female and 1 = male. The “Variable View” tab in the SPSS file of comprehensively explaining each variable and its corresponding coding.

The NHANES 2007-2012 spirometry dataset provides detailed lung function measurements for a representative sample of the U.S. population. Key variables measured include:

1. **Forced expiratory volume in 1 s (FEV_1_)**: The amount of air a person can forcefully exhale in one second after taking a deep breath.
2. **Forced vital capacity (FVC)**: The total amount of air exhaled during a forced breath.
3. **FEV_1_/FVC ratio**: A calculated ratio used to diagnose airflow obstruction and other lung disorders.
4. **Demographic information**: Age, sex, race/ethnicity (White, Black, Mexican American, Other Hispanic, multi-racial), and other sociodemographic variables are included, providing context for analyzing lung function across different groups.
5. **Body measurements**: Variables like height, weight, and Body Mass Index (BMI) are also recorded, as these factors influence lung function.
6. **Z-scores**: The dataset includes z-scores for FEV_1_, FVC, and FEV_1_/FVC ratio, which are standardized scores comparing an individual's values to population norms based on Global Lung Function Initiative (GLI) reference equations (i.e., race-neutral equations) [2].
7. **Modeling results**: Airflow obstruction, possible restriction, or mixed disorder results – as defined elsewhere [9] – were classified by the developed GAMLSS and SLR models for FEV_1_ & FVC, and, then GAMLSS and multiple linear regression for the FEV_1_/FVC ratio. This is the unique feature of this refined dataset.

All spirometry measurements meet or exceed the technical acceptability standards set by ATS and ERS in 2005 [10], ensuring the reliability and accuracy of the data. The dataset covers individuals aged 6–80, providing a wide age range for analysis of lung function across the lifespan. Additionally, the dataset facilitates comparisons across racial and ethnic groups, making it a valuable resource for understanding population-specific lung function trends and disparities.

## 4 Experimental Design, Materials and Methods

This is a cross-sectional study, as the spirometry data was collected at a single time. This snapshot captures a variety of ages without considering individual changes over time. Yet, the design allows for analysing trends and associations related to age in lung function across a large sample.

This dataset [7] includes only NHANES participants who met “A” and “B” grade acceptability standards as defined by the National Institute for Occupational Safety and Health (NIOSH). The criteria for spirometry values were as follows: for an “A” grade, three acceptable curves were required, with the largest and second-largest values within 100 ml and no more than a 50 ml difference from the last maneuver. Three acceptable curves were required for a “B” grade, with the largest and second-largest values within 150 ml, meeting the minimum criteria set by the American Thoracic Society's 2005 guidelines [10]. Based on these criteria, 16,596 subjects were retained from an initial pool of around 30,000.

The dataset included both males and females, ranging in age from 6 to 80 years, across five racial/ethnic categories (White, Black, Mexican American, Other Hispanic, and multi-racial). Since the focus was not on comparing spirometry differences across races or ethnicities, but rather on comparing the RMSE between different modeling techniques, all racial/ethnic groups were analyzed together as one pooled group.

Reference equations for FEV_1_, FVC, and the FEV_1_/FVC ratio were developed using three modeling techniques: GAMLSS and segmented linear regression (SLR) for FEV_1_ and FVC, and multiple linear regression and GAMLSS for the FEV_1_/FVC ratio. SLR was not used for the FEV_1_/FVC ratio, as no significant breakpoint was identified across the lifespan.

All analyses were performed in the R programming environment, with age, height, and weight identified as key predictors.

### 4.1 Statistical Analyses

Two primary statistical models were compared: GAMLSS and segmented linear regression. For prediction accuracy, *K*-fold cross-validation (with 10 folds) was used to estimate the root-mean-square error (RMSE) and correlation coefficients for FEV_1_, FVC, and FEV_1_/FVC. The agreement between these two models in classifying spirometric patterns (e.g., airflow obstruction, restrictive spirometry) was assessed using the unweighted Kappa statistic**.** Additionally, Bayesian Information Criterion (BIC) and Akaike Information Criterion (AIC) were employed to compare the goodness-of-fit between models, and paired *t*-tests were used to evaluate differences in z-scores produced by the models. McNemar's test was applied to assess differences in the classification of spirometry patterns, and multiple comparison corrections were made using the Benjamini-Hochberg procedure to control for false discovery rates [11].

These statistical analyses ensured a thorough comparison between the simpler segmented regression and the more complex GAMLSS models in predicting lung function across the dataset.

The GAMLSS models [12] and SLR [13] were implemented using R CRAN packages. All the R-package names are provided in the supplementary file to this data article that includes the “bare bones” R-code. Statistical significance was defined as *p <* 0.05.

## Limitations

The refined dataset, available through the Mendeley Data repository [7] has certain limitations that should be acknowledged. Firstly, only a portion of the NHANES data was included, as the dataset was restricted to individuals who successfully completed technically valid spirometry tests. As a result, the exclusion of participants who could not perform these maneuvers might have affected the outcomes, potentially leading to different conclusions if the full NHANES dataset had been analyzed. Additionally, the decision to combine racial and ethnic groups may have concealed important differences in lung function between populations, limiting the findings' relevance for specific demographic groups. Finally, since the data is cross-sectional, it does not allow for the analysis of lung function changes over time, which could provide more detailed insights into the effects of factors like growth, aging, and environmental exposures.

## Ethics Statement

Ethics approval was unnecessary as this was de-identified publicly available data from NHANES.

## CRediT authorship contribution statement

**Gerald Stanley Zavorsky:** Conceptualization, Software, Writing – original draft, Formal analysis, Methodology, Investigation, Writing – review & editing.

## Data Availability

Mendeley DataRefined NHANES 2007-2012 spirometry dataset for the comparison of segmented (piecewise) linear models to that of GAMLSS (Reference data)

## Appendix Supplementary materials

Image, application 1
