# Supplementary material for: A refined spirometry dataset for comparing segmented (piecewise) linear models to that of GAMLSS
Source: Data Brief. 2024 Oct 23;57:111062. doi: 10.1016/j.dib.2024.111062 (PMC11684394; doi:10.1016/j.dib.2024.111062)
Supplement: Supplementary file 1 [file mmc1.docx]

# **Supplement to the article:**

# **Refined NHANES 2007-2012 spirometry dataset for the comparison of segmented (piecewise) linear models to that of GAMLSS.**

^1^Gerald S. Zavorsky PhD, RRT, RPFT, FACSM

^1^Department of Physiology and Membrane Biology, University of California, Davis, Medical Center, Sacramento, California, United States.

**Gerald Zavorsky: ORCID ID:** [**https://orcid.org/0000-0002-4473-1601**](https://orcid.org/0000-0002-4473-1601)

Address for Correspondence:

Gerald S. Zavorsky, Ph.D., RRT, RPFT, FACSM

Adjunct Professor

Department of Physiology and Membrane Biology
University of California, Davis

e-mail: [gszavorsky@ucdavis.edu](mailto:gszavorsky@ucdavis.edu)

The following pages are the “bare bones” R code for the developed spirometry reference equations from the refined 2007-2012 NHANES dataset. The code for each developed reference equation is presented in the following pages:

- GAMLSS: FEV_1_/FVC ratio in Males = Page 3
- GAMLSS: FEV_1_/FVC ratio in Females = Page 5
- GAMLSS: FEV_1_ in Males = Page 7
- GAMLSS FEV_1_ in Females = Page 9
- GAMLSS FVC in Males = Page 11
- GAMLSS FVC in Females = Page 13
- Multiple linear regression FEV_1_/FVC in Males = Page 15
- Multiple Linear regression: FEV_1_/FVC in Females = Page 16
- Segmented linear regression FEV_1_ in Males = Page 17
- Segmented linear regression FEV_1_ in Females Page 19
- Segmented linear regression FVC in Males = Page 21
- Segmented linear regression FVC in Females = Page 23

Where:

FEV_1_ is forced expiratory volume in 1 s (Liters)

FVC = forced vital capacity (Liters)

FEV_1_/FVC ratio = amount of air that can be forcefully exhaled in 1 s compared to the total maximal volume of air in the lungs (i.e. vital capacity, which does not include residual volume).

The reference equations use the refined NHANES data which pool all races/ethnicities as one group since the main objective was to compare predictive accuracies between models, not to identify differences in spirometry values between races/ethnicities.

**Reference equation for the FEV_1_/FVC ratio in males using GAMLSS**

#Libraries loaded

library(gamlss); library(gamlss.dist); library(gamlss.data);library(gamlss.add); library(haven); library(writexl); library(openxlsx); library(splines); library(ggplot2); library(SemiPar);library(dplyr)

################################################################################

#FEV_1_/FVC RATIO MALES GAMLSS

# Fit a null model (intercept-only model)

null_model1 <- gamlss(Baseline_FEV1_FVC_Ratio ~ 1,

sigma.formula = ~1,

family = BCCGo,

data = Spirometry_Male)

summary(null_model1)

FEV1_FVC_Ratio<- gamlss(Baseline_FEV1_FVC_Ratio ~ log(Height) + log(Age) + cs(Age, df=5),

sigma.formula = ~ log(Age)+ cs(Age, df=5),

family = BCCGo (mu.link = "log"), data = Spirometry_Male,

nu.start = 1, nu.fix=FALSE, method=mixed(),

control = gamlss.control(trace=TRUE, maxit=1000, tol=1e-07))

summary(FEV1_FVC_Ratio)

# Get R-squared value for the model

rsq_value <- Rsq(FEV1_FVC_Ratio, type = "both")

# Extract the individual R-squared values

rsq_mean <- rsq_value$mu

rsq_sigma <- rsq_value$sigma

# Round the values to 2 decimal places

rsq_rounded_CoxSnell <- round(rsq_value$CoxSnell, 2)

# Print the rounded values

cat("Rounded CoxSnell:", rsq_rounded_CoxSnell, "\n")

# Obtain the residual sum of squares (RSS) or deviance

RSS <- deviance(FEV1_FVC_Ratio)

# Standardized residuals vs. fitted values

plot(FEV1_FVC_Ratio, what="stdres", type="response")

range(resid(FEV1_FVC_Ratio))

# Print summary and RSS

summary(FEV1_FVC_Ratio)

cat("Residual Sum of Squares (Deviance):", RSS, "\n")

# Calculate McFadden's R-squared

ll_full <- logLik(FEV1_FVC_Ratio)[1]

ll_null_model <- gamlss(Baseline_FEV1_FVC_Ratio ~ 1, data = Spirometry_Male, family = BCCGo(mu.link = "log"))

ll_null <- logLik(ll_null_model)[1]

r2_mcfadden <- 1 - (ll_full/ll_null)

# Calculate RSS for each model

residuals <- residuals(FEV1_FVC_Ratio)

RSS <- sum(residuals^2)

# Print McFadden's R-squared

print(r2_mcfadden)

#Histogram of Residuals

hist(residuals(FEV1_FVC_Ratio, type="weighted"))

#Worm Plot. It's a specific plot for GAMLSS models and is used to check if the distribution assumption is correct. The worm plot should show all points inside the confidence bands

library(gamlss.add)

wp(FEV1_FVC_Ratio)

# Influence plot. They help identify influential observations. High leverage points are those observations, if any, that are far away from the other observations in terms of predictor values.

plot(FEV1_FVC_Ratio, what="influence")

# Load necessary package

library(gamlss)

# Assuming FEV1_FVC_Ratio is your fitted model object

# Calculate centiles

centiles_FEV1_FVC_Ratio <- centiles(FEV1_FVC_Ratio, xvar = Spirometry_Male$Age, cent = c(1, 2.5, 5, 50, 95, 97.5, 99))

# Plot the term

term.plot(FEV1_FVC_Ratio, what = "mu", se = TRUE, partial = FALSE,

col.term = "black", col.se = "black", las = 1)

**Reference equation for the FEV_1_/FVC ratio in females using GAMLSS**

#Libraries loaded

library(gamlss); library(gamlss.dist); library(gamlss.data);library(gamlss.add); library(haven); library(writexl); library(openxlsx); library(splines);library(ggplot2); library(SemiPar); library(dplyr)

################################################################################

#FEV_1_/FVC RATIO FEMALES GAMLSS

# Rename file

Spirometry = NHANES_2007_2012_Only_Acceptable_Spirometry_Values

# Split data into male and female subsets

Spirometry_Male <- subset(Spirometry, Sex == 1)

Spirometry_Female <- subset(Spirometry, Sex == 0)

# Fit a null model (intercept-only model)

null_model1 <- gamlss(Baseline_FEV1_FVC_Ratio ~ 1,

sigma.formula = ~1,

family = BCCGo,

data = Spirometry_Female)

FEV1_FVC_Ratio<- gamlss(Baseline_FEV1_FVC_Ratio ~ log(Height) + log(Age) + cs(Age, df=5),

sigma.formula = ~ log(Age)+ cs(Age, df=5),

family = BCCGo (mu.link = "log"), data = Spirometry_Female,

nu.start = 1, nu.fix=FALSE, method=mixed(),

control = gamlss.control(trace=TRUE, maxit=1000, tol=1e-07))

summary(FEV1_FVC_Ratio)

# Get R-squared value for the model

rsq_value <- Rsq(FEV1_FVC_Ratio, type = "both")

# Extract the individual R-squared values

rsq_mean <- rsq_value$mu

rsq_sigma <- rsq_value$sigma

# Round the values to 2 decimal places

rsq_rounded_CoxSnell <- round(rsq_value$CoxSnell, 2)

# Print the rounded values

cat("Rounded CoxSnell:", rsq_rounded_CoxSnell, "\n")

# Obtain the residual sum of squares (RSS) or deviance

RSS <- deviance(FEV1_FVC_Ratio)

# Standardized residuals vs. fitted values

plot(FEV1_FVC_Ratio, what="stdres", type="response")

range(resid(FEV1_FVC_Ratio))

# Print summary and RSS

summary(FEV1_FVC_Ratio)

cat("Residual Sum of Squares (Deviance):", RSS, "\n")

# Calculate McFadden's R-squared

ll_full <- logLik(FEV1_FVC_Ratio)[1]

ll_null_model <- gamlss(Baseline_FEV1_FVC_Ratio ~ 1, data = Spirometry_Female, family = BCCGo(mu.link = "log"))

ll_null <- logLik(ll_null_model)[1]

r2_mcfadden <- 1 - (ll_full/ll_null)

# Calculate RSS for each model

residuals <- residuals(FEV1_FVC_Ratio)

RSS <- sum(residuals^2)

# Print McFadden's R-squared

print(r2_mcfadden)

#Histogram of Residuals

hist(residuals(FEV1_FVC_Ratio, type="weighted"))

#Worm Plot. It's a specific plot for GAMLSS models and is used to check if the distribution assumption is correct. The worm plot should show all points inside the confidence bands

wp(FEV1_FVC_Ratio)

# Influence plot. They help identify influential observations. High leverage points are those observations, if any, that are far away from the other observations in terms of predictor values.

plot(FEV1_FVC_Ratio, what="influence")

# Calculate centiles

centiles_FEV1_FVC_Ratio <- centiles(FEV1_FVC_Ratio, xvar = Spirometry_Male$Age, cent = c(1, 2.5, 5, 50, 95, 97.5, 99))

# Plot the term

term.plot(FEV1_FVC_Ratio, what = "mu", se = TRUE, partial = FALSE,

col.term = "black", col.se = "black", las = 1)

**Reference equation for FEV_1_ in males using GAMLSS**

#Libraries loaded

library(gamlss); library(gamlss.dist); library(gamlss.data);library(gamlss.add); library(haven); library(writexl); library(openxlsx); library(splines);library(ggplot2); library(SemiPar); library(dplyr)

################################################################################

#FEV_1_ MALES GAMLSS

# Rename file

Spirometry = NHANES_2007_2012_Only_Acceptable_Spirometry_Values

# Split data into male and female subsets

Spirometry_Male <- subset(Spirometry, Sex == 1)

Spirometry_Female <- subset(Spirometry, Sex == 0)

# Fit a null model (intercept-only model)

null_model1 <- gamlss(Baseline_FEV1_L ~ 1,

sigma.formula = ~1,

family = BCCGo,

data = Spirometry_Male)

summary(null_model1)

# Fitting the model using 'CS' smoothing with fixed degrees of freedom to ensure convergence

FEV1_Male <- gamlss(Baseline_FEV1_L ~ log(Height) + log(Age) + cs(Age, df=3),

sigma.formula = ~ log(Age) + cs(Age, df=3),

family = BCCGo(mu.link = "log"),

data = Spirometry_Male,

method = mixed(50, 500), # Using Conjugate Gradients method

control = gamlss.control(trace = TRUE, maxit = 500))

summary(FEV1_Male)

# Get R-squared value for the model

rsq_value <- Rsq(FEV1_Male, type = "both")

round(AIC(FEV1_Male),1)

round(BIC(FEV1_Male),1)

# Extract the individual R-squared values

rsq_mean <- rsq_value$mu

rsq_sigma <- rsq_value$sigma

# Round the values to 2 decimal places

rsq_rounded_CoxSnell <- round(rsq_value$CoxSnell, 2)

# Print the rounded values

cat("Rounded CoxSnell:", rsq_rounded_CoxSnell, "\n")

# Obtain the residual sum of squares (RSS) or deviance

RSS <- deviance(FEV1_Male)

# Calculate RSS for each model

residuals <- residuals(FEV1_Male)

RSS <- sum(residuals^2)

# Standardized residuals vs. fitted values

plot(FEV1_Male, what="stdres", type="response")

range(resid(FEV1_Male))

# Print summary and RSS

summary(FEV1_Male)

cat("Residual Sum of Squares (Deviance):", RSS, "\n")

# Calculate McFadden's R-squared

ll_full <- logLik(FEV1_Male)[1]

ll_null_model <- gamlss(Baseline_FEV1_L ~ 1, data = Spirometry_Male, family = BCCGo(mu.link = "log"))

ll_null <- logLik(ll_null_model)[1]

r2_mcfadden <- 1 - (ll_full/ll_null)

# Print McFadden's R-squared

print(r2_mcfadden)

#Histogram of Residuals

hist(residuals(FEV1_Male, type="weighted"))

#Worm Plot. It's a specific plot for GAMLSS models and is used to check if the distribution assumption is correct. The worm plot should show all points inside the confidence bands

library(gamlss.add)

wp(FEV1_Male)

# Influence plot. They help identify influential observations. High leverage points are those observations, if any, that are far away from the other observations in terms of predictor values.

plot(FEV1_Male, what="influence")

# Calculate centiles

centiles_FEV1_Male <- centiles(FEV1_Male, xvar = Spirometry_Male$Age, cent = c(1, 2.5, 5, 50, 95, 97.5, 99))

# Plot the term

term.plot(FEV1_Male, what = "mu", se = TRUE, partial = FALSE,

col.term = "black", col.se = "black", las = 1)

**Reference equation for FEV_1_ in females using GAMLSS**

#Libraries loaded

library(gamlss); library(gamlss.dist); library(gamlss.data);library(gamlss.add); library(haven); library(writexl); library(openxlsx); library(splines);library(ggplot2); library(SemiPar); library(dplyr)

################################################################################

#FEV_1_ FEMALES GAMLSS

# Rename file

Spirometry = NHANES_2007_2012_Only_Acceptable_Spirometry_Values

#Split data into male and female subsets

Spirometry_Male <- subset(Spirometry, Sex == 1)

Spirometry_Female <- subset(Spirometry, Sex == 0)

# Fit a null model (intercept-only model)

null_model1 <- gamlss(Baseline_FEV1_L ~ 1,

sigma.formula = ~1,

family = BCCGo,

data = Spirometry_Female)

summary(null_model1)

# Fitting the model using 'CS' smoothing with fixed degrees of freedom to ensure convergence

FEV1_Female <- gamlss(Baseline_FEV1_L ~ log(Height) + log(Age) + cs(Age, df=3),

sigma.formula = ~ log(Age) +cs(Age, df=3),

family = BCCGo (mu.link = "log"), data = Spirometry_Female,

nu.start = 1, nu.fix=FALSE, method=mixed(50, 500),

control = gamlss.control(trace=TRUE, maxit=500, tol=1e-07))

summary(FEV1_Female)

# Get R-squared value for the model

rsq_value <- Rsq(FEV1_Female, type = "both")

# Extract the individual R-squared values

rsq_mean <- rsq_value$mu

rsq_sigma <- rsq_value$sigma

# Round the values to 2 decimal places

rsq_rounded_CoxSnell <- round(rsq_value$CoxSnell, 2)

# Print the rounded values

cat("Rounded CoxSnell:", rsq_rounded_CoxSnell, "\n")

# Obtain the residual sum of squares (RSS) or deviance

RSS <- deviance(FEV1_Female)

# Standardized residuals vs. fitted values

plot(FEV1_Female, what="stdres", type="response")

range(resid(FEV1_Female))

# Print summary and RSS

summary(FEV1_Female)

cat("Residual Sum of Squares (Deviance):", RSS, "\n")

# Calculate McFadden's R-squared

ll_full <- logLik(FEV1_Female)[1]

ll_null_model <- gamlss(Baseline_FEV1_L ~ 1, data = Spirometry_Female, family = BCCGo(mu.link = "log"))

ll_null <- logLik(ll_null_model)[1]

r2_mcfadden <- 1 - (ll_full/ll_null)

# Print McFadden's R-squared

print(r2_mcfadden)

#Histogram of Residuals

hist(residuals(FEV1_Female, type="weighted"))

#Worm Plot. It's a specific plot for GAMLSS models and is used to check if the distribution assumption is correct. The worm plot should show all points inside the confidence bands

library(gamlss.add)

wp(FEV1_Female)

# Influence plot. They help identify influential observations. High leverage points are those observations, if any, that are far away from the other observations in terms of predictor values.

plot(FEV1_Female, what="influence")

# Calculate centiles

centiles_FEV1 <- centiles(FEV1_Female, xvar = Spirometry_Female$Age, cent = c(1, 2.5, 5, 50, 95, 97.5, 99))

# Plot the term

term.plot(FEV1_Female, what = "mu", se = TRUE, partial = FALSE,

col.term = "black", col.se = "black", las = 1)

**Reference equation for FVC in males using GAMLSS**

#Libraries loaded

library(gamlss); library(gamlss.dist); library(gamlss.data);library(gamlss.add); library(haven); library(writexl); library(openxlsx); library(splines);library(ggplot2); library(SemiPar); library(dplyr)

################################################################################

#FVC MALES GAMLSS

# Rename file

Spirometry = NHANES_2007_2012_Only_Acceptable_Spirometry_Values

#Split data into male and female subsets

Spirometry_Male <- subset(Spirometry, Sex == 1)

Spirometry_Female <- subset(Spirometry, Sex == 0)

# Fit a null model (intercept-only model)

null_model1 <- gamlss(Baseline_FVC_L ~ 1,

sigma.formula = ~1,

family = BCCGo,

data = Spirometry_Male)

summary(null_model1)

# Fitting the model using 'CS' smoothing with fixed degrees of freedom to ensure convergence

FVC_Male <- gamlss(Baseline_FVC_L ~ log(Height) + log(Age) + cs(Age, df=3),

sigma.formula = ~ log(Age) + cs(Age,df=3),

family = BCCGo(mu.link = "log"),

data = Spirometry_Male,

method = mixed(50, 500), # Using Conjugate Gradients method

control = gamlss.control(trace = TRUE, maxit = 500))

# Display the summary of the model

summary(FVC_Male)

# Get R-squared value for the model

rsq_value <- Rsq(FVC_Male, type = "both")

# Extract the individual R-squared values

rsq_mean <- rsq_value$mu

rsq_sigma <- rsq_value$sigma

# Round the values to 2 decimal places

rsq_rounded_CoxSnell <- round(rsq_value$CoxSnell, 2)

# Print the rounded values

cat("Rounded CoxSnell:", rsq_rounded_CoxSnell, "\n")

# Obtain the residual sum of squares (RSS) or deviance

RSS <- deviance(FVC_Male)

# Standardized residuals vs. fitted values

plot(FVC_Male, what="stdres", type="response")

range(resid(FVC_Male))

# Print summary and RSS

summary(FVC_Male)

cat("Residual Sum of Squares (Deviance):", RSS, "\n")

#Histogram of Residuals

hist(residuals(FVC_Male, type="weighted"))

#Worm Plot. It's a specific plot for GAMLSS models and is used to check if the distribution assumption is correct. The worm plot should show all points inside the confidence bands

library(gamlss.add)

wp(FVC_Male)

# Influence plot. They help identify influential observations. High leverage points are those observations, if any, that are far away from the other observations in terms of predictor values.

plot(FVC_Male, what="influence")

# Calculate centiles

centiles_FVC_Male <- centiles(FVC_Male, xvar = Spirometry_Male$Age, cent = c(1, 2.5, 5, 50, 95, 97.5, 99))

# Plot the term

term.plot(FVC_Male, what = "mu", se = TRUE, partial = FALSE,

col.term = "black", col.se = "black", las = 1)

**Reference equation for FVC in females using GAMLSS**

#Libraries loaded

library(gamlss); library(gamlss.dist); library(gamlss.data);library(gamlss.add); library(haven); library(writexl); library(openxlsx); library(splines);library(ggplot2); library(SemiPar);library(dplyr)

################################################################################

#FVC FEMALES GAMLSS

# Rename file

Spirometry = NHANES_2007_2012_Only_Acceptable_Spirometry_Values

#Split data into male and female subsets

Spirometry_Male <- subset(Spirometry, Sex == 1)

Spirometry_Female <- subset(Spirometry, Sex == 0)

# Fit a null model (intercept-only model)

null_model1 <- gamlss(Baseline_FVC_L ~ 1,

sigma.formula = ~1,

family = BCCGo,

data = Spirometry_Female)

summary(null_model1)

# Fitting the model using 'CS' smoothing with fixed degrees of freedom to ensure convergence

FVC_Female <- gamlss(Baseline_FVC_L ~ log(Height) + log(Age) + cs(Age, df=3),

sigma.formula = ~ log(Age) + cs(Age,df=3),

family = BCCGo(mu.link = "log"),

data = Spirometry_Female,

method = mixed(50, 500), # Using Conjugate Gradients method

control = gamlss.control(trace = TRUE, maxit = 500))

# Display the summary of the model

summary(FVC_Female)

# Get R-squared value for the model

rsq_value <- Rsq(FVC_Female, type = "both")

# Extract the individual R-squared values

rsq_mean <- rsq_value$mu

rsq_sigma <- rsq_value$sigma

# Round the values to 2 decimal places

rsq_rounded_CoxSnell <- round(rsq_value$CoxSnell, 2)

# Print the rounded values

cat("Rounded CoxSnell:", rsq_rounded_CoxSnell, "\n")

# Obtain the residual sum of squares (RSS) or deviance

RSS <- deviance(FVC_Female)

# Standardized residuals vs. fitted values

plot(FVC_Female, what="stdres", type="response")

range(resid(FVC_Female))

# Print summary and RSS

summary(FVC_Female)

cat("Residual Sum of Squares (Deviance):", RSS, "\n")

#Histogram of Residuals

hist(residuals(FVC_Female, type="weighted"))

#Worm Plot. It's a specific plot for GAMLSS models and is used to check if the distribution assumption is correct. The worm plot should show all points inside the confidence bands

library(gamlss.add)

wp(FVC_Female)

# Influence plot. They help identify influential observations. High leverage points are those observations, if any, that are far away from the other observations in terms of predictor values.

plot(FVC_Female, what="influence")

# Calculate centiles

centiles_FVC_Female <- centiles(FVC_Female, xvar = Spirometry_Female$Age, cent = c(1, 2.5, 5, 50, 95, 97.5, 99))

# Plot the term

term.plot(FVC_Female, what = "mu", se = TRUE, partial = FALSE,

col.term = "black", col.se = "black", las = 1)

**Reference equation for the FEV_1_/FVC ratio in females using stepwise multiple linear regression**

# Load necessary libraries

library(lmtest); library(MASS); library(boot);library(knitr);library(performance); library(patchwork); library(relaimpo); library(ggplot2); library(writexl); library(openxlsx); library(haven); library(dplyr)

#################################################################################

#Rename file

Spirometry = NHANES_2007_2012_Only_Acceptable_Spirometry_Values

#Rename file

Spirometry = NHANES_2007_2012_Only_Acceptable_Spirometry_Values

#Split data into male and female subsets

Spirometry_Male <- subset(Spirometry, Sex == 1)

Spirometry_Female <- subset(Spirometry, Sex == 0)

# FEV_1_/FVC ratio FEMALES

# Get the number of subjects

num_subjects <- nrow(Spirometry)

# Define the full model with all predictors

Predicted_FEV1_FVC_Ratio <- lm(Baseline_FEV1_FVC_Ratio ~ Age + Age2 + Age3 + Height + Height2 + Age_Height + Age_Height2 + Weight, data = Spirometry_Female)

# Perform stepwise regression using BIC

best_model <- stepAIC(Predicted_FEV1_FVC_Ratio, k = log(nrow(Spirometry)), direction = "both", trace = FALSE)

# Summary of the best model

summary_best_model <- summary(best_model)

summary_best_model

**Reference equation for the FEV_1_/FVC ratio in males using stepwise multiple linear regression**

# Load necessary libraries

library(lmtest); library(MASS); library(boot);library(knitr);library(performance); library(patchwork); library(relaimpo); library(ggplot2); library(writexl); library(openxlsx); library(haven); library(dplyr)

#################################################################################

#Rename file

Spirometry = NHANES_2007_2012_Only_Acceptable_Spirometry_Values

#FEV1/FVC Males

# Get the number of subjects

num_subjects <- nrow(Spirometry_Male)

# Define the full model with all predictors

Predicted_FEV1_FVC_Ratio <- lm(Baseline_FEV1_FVC_Ratio ~ Age + Age2 + Age3 + Height + Height2 + Age_Height + Age_Height2

+ Weight, data = Spirometry_Male)

# Perform stepwise regression using BIC

best_model <- stepAIC(Predicted_FEV1_FVC_Ratio, k = log(nrow(Spirometry_Male)), direction = "both", trace = FALSE)

# Summary of the best model

summary_best_model <- summary(best_model)

summary_best_model

**Reference equation for FEV_1_ in males using segmented linear regression**

#Load libraries

library(dplyr); library(haven); library(AICcmodavg); library(mvtnorm); library(PropCIs); library(glmnet);

library(segmented); library(relaimpo)

Spirometry = NHANES_2007_2012_Only_Acceptable_Spirometry_Values

#################################################################################

# Split data into male and female subsets

Spirometry_Male <- subset(Spirometry, Sex == 1)

Spirometry_Female <- subset(Spirometry, Sex == 0)

Predicted_FEV1_L <- lm(Baseline_FEV1_L~Age2+Height+Height2+Weight, data=Spirometry_Male)

FEV1<-segmented(Predicted_FEV1_L, seg.Z =~Age2, psi=22^2)

# Obtain predicted values

predicted_values <- predict(FEV1)

# Calculate residuals

residuals <- Spirometry_Male$Baseline_FEV1_L - predicted_values

# Calculate sum of squared residuals

ssr <- sum(residuals^2)

summary(FEV1, var.diff = TRUE)

intercept(FEV1)

slope(FEV1)

(summary(FEV1)$psi)^0.5

round(AIC(FEV1),1)

round(BIC(FEV1), 1)

print(ssr) # Print the SSR

#95% CI

confint.seg <- function(obj){

tmp <- summary(obj)

tmp_coef <- tmp$coefficients

est <- tmp_coef[, "Estimate"]

se <- tmp_coef[, "Std. Error"]

ci <- cbind(est - (1.96*se),est + (1.96*se))

results <- as.data.frame(tmp_coef)

results$ci.lower <- ci[, 1]

results$ci.upper <- ci[, 2]

return(results)

}

sqrt(confint(FEV1))

confint.seg(FEV1)

round(confint.seg(FEV1), 5)

# Calculate residuals

residuals <- Spirometry_Male$Baseline_FEV1_L - predicted_values

# Calculate sum of squared residuals

ssr <- sum(residuals^2)

# Print the SSR

print(ssr)

#Variable R^2^ contribution: Relative importance of predictors in a Segmented regression model

#linear model context ignoring the potential breakpoints.

full_model <- lm(Baseline_FEV1_L~Age2+Height+Height2+Weight, data=Spirometry_Male)

FEV1 <-segmented(Predicted_FEV1_L, seg.Z =~Age2, psi=22^2)

# Calculate relative importance

importance <- calc.relimp(full_model, type="lmg")

# Convert to percentages and round to one decimal place

importance_perc <- round(importance$lmg * 100, 1)

# Order the results in descending order

ordered_importance <- sort(importance_perc, decreasing = TRUE)

# Convert to a data frame for better presentation

importance_df <- data.frame(Variable = names(ordered_importance),

R2_Contribution = ordered_importance)

# Display results without row names

print(importance_df, row.names = FALSE)

# Bootstrapping

fixedlmg <- booteval.relimp(boot.relimp(full_model, b = 1000, fixed = TRUE), bty = "perc", level = 0.95)

randomlmg <- booteval.relimp(boot.relimp(full_model, b = 1000), bty = "perc", level = 0.95)

output <- rbind(fixedlmg$lmg.lower, fixedlmg$lmg.upper, randomlmg$lmg.lower, randomlmg$lmg.upper)

output <- as.matrix(t(output))

# Convert to percentages and round to the first decimal

output <- round(output * 100, 1)

colnames(output) <- c("fixed.lower", "fixed.upper", "random.lower", "random.upper")

# Filter out FEV1 from the names and assign row names to output

filtered_names <- fixedlmg$namen[fixedlmg$namen != "FEV1"]

rownames(output) <- filtered_names[1:nrow(output)]

output

**Reference equation for FEV_1_ in females using segmented linear regression**

#Load libraries

library(dplyr); library(haven); library(AICcmodavg); library(mvtnorm); library(PropCIs); library(glmnet);

library(segmented); library(relaimpo)

Spirometry = NHANES_2007_2012_Only_Acceptable_Spirometry_Values

##################################################################################

# Split data into male and female subsets

Spirometry_Male <- subset(Spirometry, Sex == 1)

Spirometry_Female <- subset(Spirometry, Sex == 0)

Predicted_FEV1_L <- lm(Baseline_FEV1_L~Age2+Height2+Weight+Age_Height2, data=Spirometry_Female)

FEV1<-segmented(Predicted_FEV1_L, seg.Z =~Age2, psi=22^2)

# Obtain predicted values

predicted_values <- predict(FEV1)

# Calculate residuals

residuals <- Spirometry_Female$Baseline_FEV1_L - predicted_values

# Calculate sum of squared residuals

ssr <- sum(residuals^2)

summary(FEV1, var.diff = TRUE)

intercept(FEV1)

slope(FEV1)

(summary(FEV1)$psi)^0.5

round(AIC(FEV1),1)

round(BIC(FEV1), 1)

print(ssr) # Print the SSR

#95% CI

confint.seg <- function(obj){

tmp <- summary(obj)

tmp_coef <- tmp$coefficients

est <- tmp_coef[, "Estimate"]

se <- tmp_coef[, "Std. Error"]

ci <- cbind(est - (1.96*se),est + (1.96*se))

results <- as.data.frame(tmp_coef)

results$ci.lower <- ci[, 1]

results$ci.upper <- ci[, 2]

return(results)

}

sqrt(confint(FEV1))

confint.seg(FEV1)

round(confint.seg(FEV1), 5)

# Calculate residuals

residuals <- Spirometry_Female$Baseline_FEV1_L - predicted_values

# Calculate sum of squared residuals

ssr <- sum(residuals^2)

# Print the SSR

print(ssr)

#Variable R^2^ contribution: Relative importance of predictors in a Segmented regression model

#linear model context ignoring the potential breakpoints.

full_model <- lm(Baseline_FEV1_L~Age2+Height2+Weight+Age_Height2, data=Spirometry_Female)

FEV1 <-segmented(Predicted_FEV1_L, seg.Z =~Age2, psi=22^2)

# Calculate relative importance

importance <- calc.relimp(full_model, type="lmg")

# Convert to percentages and round to one decimal place

importance_perc <- round(importance$lmg * 100, 1)

# Order the results in descending order

ordered_importance <- sort(importance_perc, decreasing = TRUE)

# Convert to a data frame for better presentation

importance_df <- data.frame(Variable = names(ordered_importance),

R2_Contribution = ordered_importance)

# Display results without row names

print(importance_df, row.names = FALSE)

# Bootstrapping

fixedlmg <- booteval.relimp(boot.relimp(full_model, b = 1000, fixed = TRUE), bty = "perc", level = 0.95)

randomlmg <- booteval.relimp(boot.relimp(full_model, b = 1000), bty = "perc", level = 0.95)

output <- rbind(fixedlmg$lmg.lower, fixedlmg$lmg.upper, randomlmg$lmg.lower, randomlmg$lmg.upper)

output <- as.matrix(t(output))

# Convert to percentages and round to the first decimal

output <- round(output * 100, 1)

colnames(output) <- c("fixed.lower", "fixed.upper", "random.lower", "random.upper")

# Filter out FEV1 from the names and assign row names to output

filtered_names <- fixedlmg$namen[fixedlmg$namen != "FEV1"]

rownames(output) <- filtered_names[1:nrow(output)]

output

**Reference equation for FVC in males using segmented linear regression**

#Load libraries

library(dplyr); library(haven); library(AICcmodavg); library(mvtnorm); library(PropCIs); library(glmnet);

library(segmented); library(relaimpo)

Spirometry = NHANES_2007_2012_Only_Acceptable_Spirometry_Values

##########################################################################

# Split data into male and female subsets

Spirometry_Male <- subset(Spirometry, Sex == 1)

Spirometry_Female <- subset(Spirometry, Sex == 0)

# MALES FVC (L) – Segmented

Predicted_FVC_L <- lm(Baseline_FVC_L~Age2+Weight+Height+Height2, data=Spirometry_Male)

FVC <-segmented(Predicted_FVC_L, seg.Z =~Age2, psi=22^2)

# Obtain predicted values

predicted_values <- predict(FVC)

# Calculate residuals

residuals <- Spirometry_Male$Baseline_FVC_L - predicted_values

# Calculate sum of squared residuals

ssr <- sum(residuals^2)

summary(FVC, var.diff = TRUE)

intercept(FVC)

slope(FVC)

(summary(FVC)$psi)^0.5

round(AIC(FVC),1)

round(BIC(FVC), 1)

print(ssr) # Print the SSR

#95% CI

confint.seg <- function(obj){

tmp <- summary(obj)

tmp_coef <- tmp$coefficients

est <- tmp_coef[, "Estimate"]

se <- tmp_coef[, "Std. Error"]

ci <- cbind(est - (1.96*se),est + (1.96*se))

results <- as.data.frame(tmp_coef)

results$ci.lower <- ci[, 1]

results$ci.upper <- ci[, 2]

return(results)

}

sqrt(confint(FVC))

confint.seg(FVC)

round(confint.seg(FVC), 5)

# Calculate residuals

residuals <- Spirometry_Male$Baseline_FVC_L - predicted_values

# Calculate sum of squared residuals

ssr <- sum(residuals^2)

# Print the SSR

print(ssr)

#Variable R^2^ contribution: Relative importance of predictors in a Segmented regression model

#linear model context ignoring the potential breakpoints.

full_model <- lm(Baseline_FVC_L~Age2+Weight+Height+Height2, data=Spirometry_Male)

FVC <-segmented(Predicted_FVC_L, seg.Z =~Age2, psi=22^2)

# Calculate relative importance

importance <- calc.relimp(full_model, type="lmg")

# Convert to percentages and round to one decimal place

importance_perc <- round(importance$lmg * 100, 1)

# Order the results in descending order

ordered_importance <- sort(importance_perc, decreasing = TRUE)

# Convert to a data frame for better presentation

importance_df <- data.frame(Variable = names(ordered_importance),

R2_Contribution = ordered_importance)

# Display results without row names

print(importance_df, row.names = FALSE)

# Bootstrapping

fixedlmg <- booteval.relimp(boot.relimp(full_model, b = 1000, fixed = TRUE), bty = "perc", level = 0.95)

randomlmg <- booteval.relimp(boot.relimp(full_model, b = 1000), bty = "perc", level = 0.95)

output <- rbind(fixedlmg$lmg.lower, fixedlmg$lmg.upper, randomlmg$lmg.lower, randomlmg$lmg.upper)

output <- as.matrix(t(output))

# Convert to percentages and round to the first decimal

output <- round(output * 100, 1)

colnames(output) <- c("fixed.lower", "fixed.upper", "random.lower", "random.upper")

# Filter out FVC from the names and assign row names to output

filtered_names <- fixedlmg$namen[fixedlmg$namen != "FVC"]

rownames(output) <- filtered_names[1:nrow(output)]

output

**Reference equation for FVC in females using segmented linear regression**

#Load libraries

library(dplyr); library(haven); library(AICcmodavg); library(mvtnorm); library(PropCIs); library(glmnet);

library(segmented); library(relaimpo)

Spirometry = NHANES_2007_2012_Only_Acceptable_Spirometry_Values

##########################################################################

# Split data into male and female subsets

Spirometry_Male <- subset(Spirometry, Sex == 1)

Spirometry_Female <- subset(Spirometry, Sex == 0)

Predicted_FVC_L <- lm(Baseline_FVC_L~Age2+Height+Height2+Weight, data=Spirometry_Female)

FVC <-segmented(Predicted_FVC_L, seg.Z =~Age2, psi=22^2)

# Obtain predicted values

predicted_values <- predict(FVC)

# Calculate residuals

residuals <- Spirometry_Female$Baseline_FVC_L - predicted_values

# Calculate sum of squared residuals

ssr <- sum(residuals^2)

summary(FVC, var.diff = TRUE)

intercept(FVC)

slope(FVC)

(summary(FVC)$psi)^0.5

round(AIC(FVC),1)

round(BIC(FVC), 1)

print(ssr) # Print the SSR

#95% CI

confint.seg <- function(obj){

tmp <- summary(obj)

tmp_coef <- tmp$coefficients

est <- tmp_coef[, "Estimate"]

se <- tmp_coef[, "Std. Error"]

ci <- cbind(est - (1.96*se),est + (1.96*se))

results <- as.data.frame(tmp_coef)

results$ci.lower <- ci[, 1]

results$ci.upper <- ci[, 2]

return(results)

}

sqrt(confint(FVC))

confint.seg(FVC)

round(confint.seg(FVC), 5)

# Calculate residuals

residuals <- Spirometry_Female$Baseline_FVC_L - predicted_values

# Calculate sum of squared residuals

ssr <- sum(residuals^2)

# Print the SSR

print(ssr)

#Variable R^2^ contribution: Relative importance of predictors in a Segmented regression model

#linear model context ignoring the potential breakpoints.

full_model <- lm(Baseline_FVC_L~Age2+Weight+Height+Height2, data=Spirometry_Female)

FVC <-segmented(Predicted_FVC_L, seg.Z =~Age2, psi=22^2)

# Calculate relative importance

importance <- calc.relimp(full_model, type="lmg")

# Convert to percentages and round to one decimal place

importance_perc <- round(importance$lmg * 100, 1)

# Order the results in descending order

ordered_importance <- sort(importance_perc, decreasing = TRUE)

# Convert to a data frame for better presentation

importance_df <- data.frame(Variable = names(ordered_importance),

R2_Contribution = ordered_importance)

# Display results without row names

print(importance_df, row.names = FALSE)

# Bootstrapping

fixedlmg <- booteval.relimp(boot.relimp(full_model, b = 1000, fixed = TRUE), bty = "perc", level = 0.95)

randomlmg <- booteval.relimp(boot.relimp(full_model, b = 1000), bty = "perc", level = 0.95)

output <- rbind(fixedlmg$lmg.lower, fixedlmg$lmg.upper, randomlmg$lmg.lower, randomlmg$lmg.upper)

output <- as.matrix(t(output))

# Convert to percentages and round to the first decimal

output <- round(output * 100, 1)

colnames(output) <- c("fixed.lower", "fixed.upper", "random.lower", "random.upper")

# Filter out FVC from the names and assign row names to output

filtered_names <- fixedlmg$namen[fixedlmg$namen != "FVC"]

rownames(output) <- filtered_names[1:nrow(output)]

output
